# Aquaporin 4-Specific T Cells in Neuromyelitis Optica Exhibit a Th17 Bias and Recognize *Clostridium* ABC Transporter

**DOI:** 10.1002/ana.23651

**Published:** 2012-07-17

**Authors:** Michel Varrin-Doyer, Collin M Spencer, Ulf Schulze-Topphoff, Patricia A Nelson, Robert M Stroud, Bruce A C Cree, Scott S Zamvil

**Affiliations:** 1 Department of Neurology, University of California at San FranciscoSan Francisco, CA; 2 Department of Biochemistry, University of California at San FranciscoSan Francisco, CA

## Abstract

**Objective:**

Aquaporin 4 (AQP4)-specific autoantibodies in neuromyelitis optica (NMO) are immunoglobulin (Ig)G1, a T cell-dependent Ig subclass, indicating that AQP4-specific T cells participate in NMO pathogenesis. Our goal was to identify and characterize AQP4-specific T cells in NMO patients and healthy controls (HC).

**Methods:**

Peripheral blood T cells from NMO patients and HC were examined for recognition of AQP4 and production of proinflammatory cytokines. Monocytes were evaluated for production of T cell-polarizing cytokines and expression of costimulatory molecules.

**Results:**

T cells from NMO patients and HC proliferated to intact AQP4 or AQP4 peptides (p11–30, p21–40, p61–80, p131–150, p156–170, p211–230, and p261–280). T cells from NMO patients demonstrated greater proliferation to AQP4 than those from HC, and responded most vigorously to p61–80, a naturally processed immunodominant determinant of intact AQP4. T cells were CD4^+^, and corresponding to association of NMO with human leukocyte antigen (HLA)-DRB1*0301 and DRB3, AQP4 p61–80-specific T cells were HLA-DR restricted. The T-cell epitope within AQP4 p61–80 was mapped to 63–76, which contains 10 residues with 90% homology to a sequence within *Clostridium perfringens* adenosine triphosphate-binding cassette (ABC) transporter permease. T cells from NMO patients proliferated to this homologous bacterial sequence, and cross-reactivity between it and self-AQP4 was observed, supporting molecular mimicry. In NMO, AQP4 p61–80-specific T cells exhibited Th17 polarization, and furthermore, monocytes produced more interleukin 6, a Th17-polarizing cytokine, and expressed elevated CD40 and CD80 costimulatory molecules, suggesting innate immunologic dysfunction.

**Interpretation:**

AQP4-specific T-cell responses are amplified in NMO, exhibit a Th17 bias, and display cross-reactivity to a protein of an indigenous intestinal bacterium, providing new perspectives for investigating NMO pathogenesis. ANN NEUROL 2012;

Neuromyelitis optica (NMO) is a rare, disabling, sometimes fatal, central nervous system (CNS) demyelinating disease characterized by severe attacks of optic neuritis and transverse myelitis.^1^ NMO is considered to be primarily a humoral autoimmune disease, as a majority of NMO patients develop autoantibodies (NMO immunoglobulin [Ig]G) against aquaporin 4 (AQP4),^2^ the predominant CNS water channel, which is abundantly expressed on astrocytes. AQP4-specific antibodies in NMO serum are IgG1, a subclass of mature IgG that requires help from T cells,^3^ indicating that AQP4-specific CD4^+^ T cells participate in the genesis of this adaptive humoral response. Passive transfer of AQP4-specific antibodies alone did not produce CNS pathology, but did promote development of NMO-like lesions in recipient animals when CNS inflammation was induced by myelin-specific T cells.^4^, ^5^ T cells are detected within active NMO lesions.^6^ Further, NMO lesions are characterized by an abundance of eosinophils and neutrophils, and elevated levels of IL-17 have been associated with NMO,^7^ suggesting involvement of Th17 cells. However, as no previous studies have identified or characterized proliferative AQP4-specific T cells in NMO patients, their potential role in NMO pathogenesis is largely unknown.

In this report, we first identified peripheral blood T cells from NMO patients and healthy controls (HC) that proliferated in response to discrete AQP4 peptides or intact AQP4. T cells from NMO patients demonstrated greater proliferation to this autoantigen than those from HC, and responded most frequently to p61–80. After defining the p61–80 core T-cell determinant, residues 63–76, we conducted a homology search with known microbes. We discovered that AQP4 p63–76 contains strong homology to aa 204–217 of an adenosine triphosphate-binding cassette (ABC) transporter permease of *Clostridium perfringens*, a bacterial species that contains both commensal and pathogenic strains for humans. T cells from NMO patients responded to the homologous ABC transporter peptide and exhibited cross-reactivity between this foreign antigen and AQP4 p63–76, findings that support molecular mimicry. When compared to HC, AQP4 p61–80-specific T cells from NMO patients exhibited Th17 polarization. Monocytes from NMO patients produced significantly higher levels of the Th17-polarizing cytokine interleukin (IL)-6, suggesting that immunologic dysfunction in NMO may also include the innate immune compartment. Collectively, our findings establish that AQP4-specific proliferative T cells exist, and support a Th17 bias in the adaptive immune response in NMO. Our demonstration of T-cell molecular mimicry may stimulate further evaluation of the potential role of the *Clostridium* species in NMO pathogenesis.

## Patients and Methods

### Patients

Fifteen NMO patients (12 females and 3 males, aged 44.3 ± 13.8 years) fulfilling Mayo Clinic diagnostic criteria^8^ and 9 HC (5 females and 4 males, aged 40.8 ± 10.7 years) were recruited from the University of California at San Francisco (UCSF) Multiple Sclerosis Center. A majority of NMO patients had been treated with rituximab,^9^ and none had been treated with azathioprine, mycophenolate mofetil, cyclophosphamide, or other immunosuppressive medications. None of the patients had received steroids within 2 months preceding blood draws. Blood was collected by venipuncture. This study was approved by the UCSF Committee on Human Research (Protocol # 10-00650), and written informed consent was obtained from subjects prior to enrollment.

### T-Cell Proliferation Assays

Peripheral blood mononuclear cells (PBMC) were isolated by density gradient centrifugation over Ficoll (Ficoll-Paque PLUS; GE Healthcare, Milwaukee, WI) according to the manufacturer's instructions. T-cell proliferation was evaluated by [^3^H]thymidine incorporation or 5,6-carboxylfluorescein diacetate succinimidyl ester (CFSE) dilution assays. In thymidine incorporation assays, PBMC were cultured with antigens in 96-well plates at either 1 × 10^5^ cells (AQP4 pools, in at least 10 wells) or 5 × 10^5^ cells (individual peptides, in duplicate) per well for 6 days. Cultures were then pulsed with [^3^H]thymidine and harvested 18 hours later. Positive wells were defined as having counts per minute (cpm) values greater than control cpm average values + 3 standard deviations or stimulation index (SI) >2. Alternatively, PBMC were stained with 0.5μM CFSE (Invitrogen, Carlsbad, CA), according to the manufacturer's instructions. Cells were cultured in the presence of antigens for 10 days. T-cell proliferation was assessed by flow cytometric evaluation of CFSE dilution. Proliferation was expressed as the cell division index (defined as the number of CFSE^low^ T cells cultured with antigen/number of CFSE^low^ T cells without antigen). In all cases, culture medium consisted of X-VIVO 15 (Lonza, Walkersville, MD) supplemented with penicillin (100U/ml) and streptomycin (0.1mg/ml).

### Antigens

Peptides were synthesized by Genemed Synthesis (San Antonio, TX) with purity >95% by high-performance liquid chromatography analysis. Overlapping AQP4 20-mer peptides were offset by 10 amino acids (Supplementary Table). Peptides corresponding to certain hydrophobic AQP4 sequences were synthesized in overlapping 15-mer peptide pairs. Truncated peptides within the 61–80 region (p61–78 GTEKPLPVDMVLISLCFG; p61–76 GTEKPLPVDMVLISLC; p61–74 GTEKPLPVDMVLIS; p61–72 GTEKPLPVDMVL; p63–80 EKPLPVDMVLISLC FGLS; p65–80 PLPVDMVLISLCFGLS; p67–80 PVDMVL ISLCFGLS; p69–80 DMVLISLCFGLS), AQP4 p63–76 (EKPLPVDMVLISLC), and bacterial peptide ABC-transporter permease (TP) p204–217 (FIILPVSMVLISLV) were as quoted. Full-length recombinant human (rh) AQP4 (1–323) was expressed in *Pichia pastoris* and purified as described.^10^ Tetanus toxoid was obtained from List Biological Laboratories (Campbell, CA).

### Flow Cytometry Analysis

Single-cell suspensions were incubated with human serum to prevent nonspecific antibody binding, then stained with antibodies against CD3, CD4, CD8, CD25, major histocompatibility complex (MHC) class II, CD40, CD80, and CD86 (eBioscience, San Diego, CA and BD Biosciences, Mississauga, ON, Canada). Intracellular cytokine production by CD4^+^ T cells and antigen-presenting cells (APC) was analyzed by monitoring the expression of interferon (IFN)-γ, IL-17, IL-6, IL-1β, and IL-10 (1:100) (eBioscience). Foxp3 staining was performed according to the manufacturer's protocol (eBioscience). For intracellular cytokine staining, T cells were stimulated with phorbol 12-myristate 13-acetate (50ng/ml) plus ionomycin (500ng/ml) in the presence of GolgiStop (1μl/ml) (BD Biosciences). CD14^+^ cells were stimulated with lipopolysaccharide (LPS; 1μg/ml; Sigma-Aldrich, St Louis, MO) for 4 or 20 hours in the presence of GolgiStop. Cells were analyzed by flow cytometry on a FACS Canto flow cytometer (BD Biosciences).

### Blocking of HLA Alleles with Antibodies

Inhibition of the proliferation of PBMC to AQP4 p61–80 and rhAQP4 was studied by using mouse monoclonal anti–HLA-DR (clone G46-6; BD Biosciences), anti–HLA-DQ (clone HG-38; Abcam, Cambridge, MA), anti–HLA-DP (clone B7/21; Abcam), and isotype control (clone G155-178; BD Biosciences). Antibodies (1μg/ml) were added to CFSE-stained PBMC cultures 1 hour before addition of antigens.

### Antigen Recall Experiments

PBMC were initially stimulated with antigens. After 10 days, cells were restimulated with rhAQP4 (5μg/ml), AQP4 peptides, or bacterial peptide (10μg/ml), in the presence of irradiated autologous APC. Following 3 days of stimulation, cultures were pulsed with [^3^H]thymidine and harvested 18 hours later. SI was calculated by dividing cpm in wells with antigen by cpm in control wells with no antigen for each assay test group.

### Analyses for Protein Sequence Homology and MHC Core Binding Motifs

Sequence similarities between AQP4 and other proteins were addressed using the protein–protein Basic Local Alignment Search Tool from the National Center for Biotechnology Information (NCBI). The prediction of the core binding motif within the AQP4 61–80 sequence for HLA-DRB1*0301 and HLA-DRB3*0202 was performed with netMHCII-1.1^11^ and net MHCII-2.2,^12^ programs that utilize relative affinities of identified determinants from the Immune Epitope Database.

### HLA Typing

High-resolution HLA typing was performed by the UCSF Immunogenetics and Transplantation Laboratory (UCSF Department of Surgery). The following HLA loci were analyzed using sequence-based typing: DRB1, DRB3/4/5, DQA1, DQB1, DPA1, and DPB1. Sequence ambiguities outside exon 2 were resolved.

### Statistics

**FIGURE 3:**
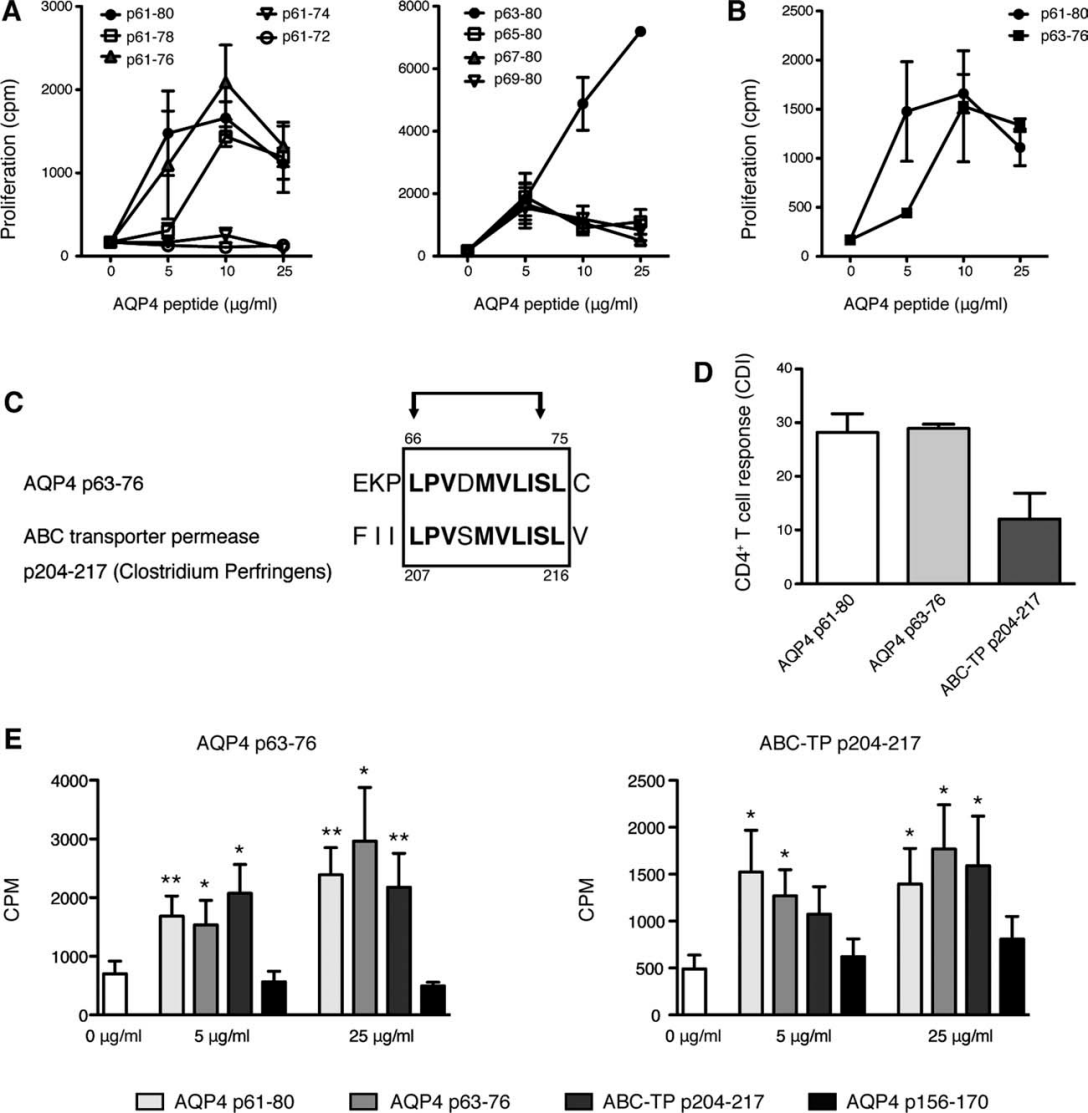
Cross-reactivity between aquaporin 4 (AQP4) p63–76 and *Clostridium* *perfringens* adenosine triphosphate-binding cassette (ABC) transporter permease (TP) p204–217. (A) The T-cell epitope within AQP4 p61–80 was mapped by testing recall proliferation of AQP4 p61–80-reactive T cells from neuromyelitis optica (NMO) patients to truncated AQP4 peptides (10μg/ml) in the presence of irradiated autologous antigen-presenting cells (APC). (B) AQP4 p63–76 appeared to contain p61–80 core determinant. Proliferation was measured by [^3^H]thymidine incorporation after 3 days. Data are representative of 3 independent experiments. (C) Sequence homology between AQP4 p63–76 and *C. perfringens* ABC-TP p204–217 was identified using the protein–protein Basic Local Alignment Search Tool from National Center for Biotechnology Information. Top bracket represents the predicted core binding motif for human leukocyte antigen (HLA)-DRB1*0301 and HLA-DRB3*0202 within AQP4 p63–76 (netMHCII-1.1 and netMHCII-2.2 programs). (D) 5,6-Carboxylfluorescein diacetate succinimidyl ester-labeled peripheral blood mononuclear cells (PBMC) from 3 NMO patients were stimulated with antigens (10μg/ml) and cultured for 10 days before evaluating proliferation by flow-activated cell sorting. (E) PBMC from 4 NMO patients were initially stimulated for 10 days with AQP4 p63–76 or ABC-TP p204–217 at 10μg/ml. Recall responses to peptides in the presence of irradiated autologous APC were evaluated by [^3^H]thymidine incorporation after 3 days. Paired *t* tests were performed to compare counts per minute (cpm) values of each antigen to cpm values of no-antigen controls, *p < 0.05, **p < 0.01. In A, B, and E, data are presented as means of duplicate or triplicate wells; error bars throughout indicate standard error of the mean. CDI = cell division index.

Statistical analysis was performed using either Prism (GraphPad Software, La Jolla, CA) or STATA (StataCorp, College Station, TX) software. The nonparametric Mann–Whitney *U* test was used to compare data. Paired *t* tests were performed to compare cpm values with antigens to control values with no antigens presented in Figure 3E. A value of *p* ≤ 0.05 was considered significant.

## Results

### T Cells from NMO Patients Recognize Discrete AQP4 Determinants and Are Restricted by HLA-DR Molecules

**FIGURE 1:**
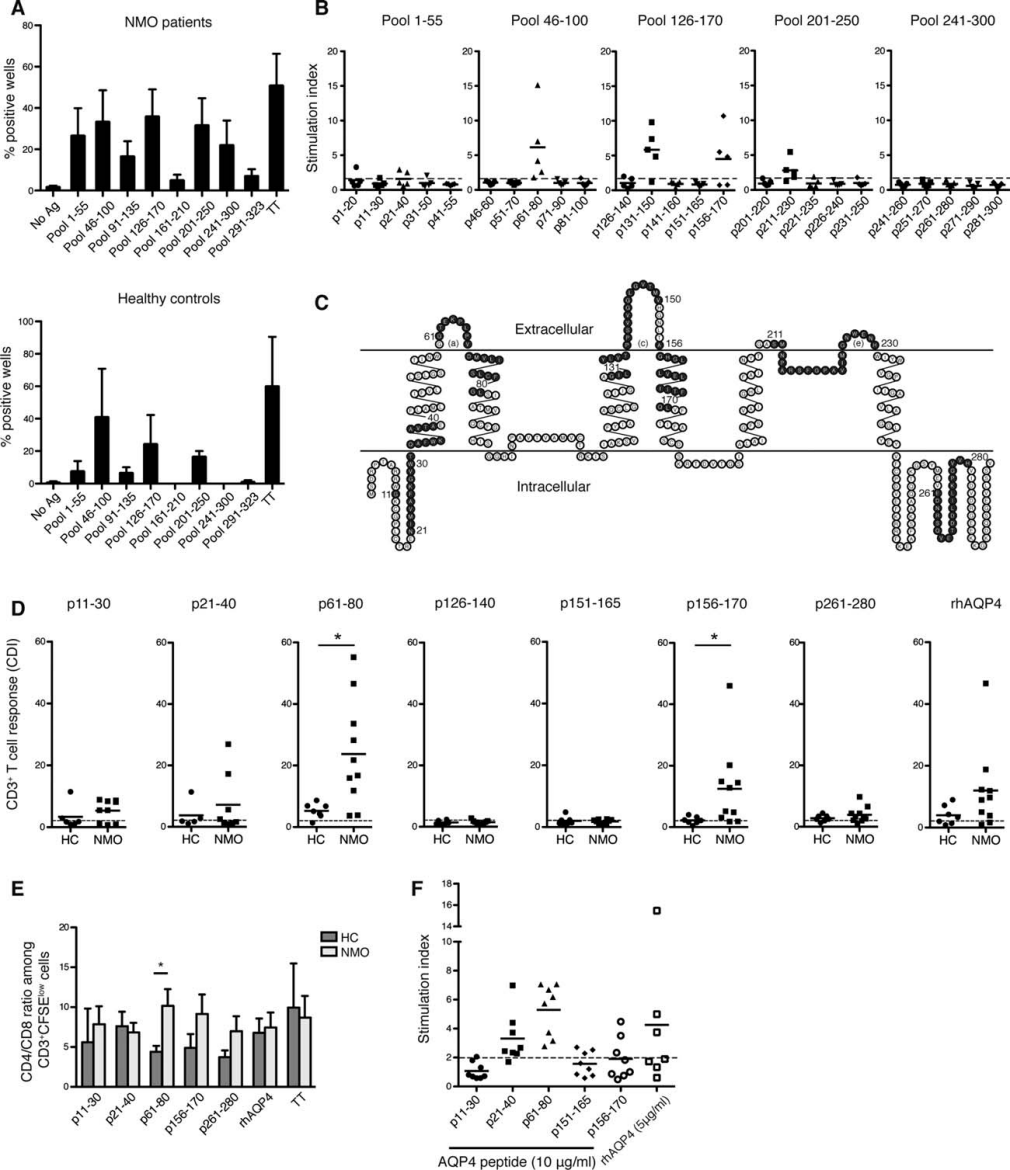
T cells from neuromyelitis optica (NMO) patients recognize discrete determinants of aquaporin 4 (AQP4). Peripheral blood mononuclear cells (PBMC) were tested for proliferation to (A) pools of AQP4 peptides (n = 8 NMO and n = 3 healthy controls [HC]) and to (B) individual AQP4 peptides identified from those pools. In A and B, PBMC were cultured for 6 days in the presence of AQP4 pools (10μg/ml) or AQP4 peptides (10μg/ml), respectively, then pulsed with [^3^H]thymidine and harvested 18 hours later. In A, positive wells were defined as values > control counts per minute average values + 3 standard deviations. (C) AQP4 determinants are represented within a human AQP4 topological diagram using TOPO2 transmembrane protein display software (http://www.sacs.ucsf.edu/TOPO2/).^50^ (D, E) PBMC were examined by 5,6-carboxylfluorescein diacetate succinimidyl ester (CFSE) dilution for proliferation to individual AQP4 peptides (10μg/ml), recombinant human (rh) AQP4 (5μg/ml), or in E, tetanus toxoid (TT; 1μg/ml) after 10 days of culture. CFSE was measured in CD3^+^, CD4^+^, and CD8^+^ T cells by flow-activated cell sorting and quantified by cell division index (CDI). CDI > 2 (*broken lines*) was considered positive. (F) Recall T-cell proliferation ([^3^H]thymidine incorporation) to individual AQP4 peptides (10μg/ml) or rhAQP4 (5μg/ml) was detected after initial stimulation with rhAQP4 (5μg/ml) for 10 days. In A and E, error bars indicate standard error of the mean; in B, D, and F, horizontal lines indicate mean values. *p < 0.05 Mann–Whitney *U* test. Ag = antigens.

In general, antigen-specific T cells recognize linear peptide fragments of 10–15 amino acids in association with MHC (HLA) proteins expressed on APC.^13^ To identify AQP4-specific T cells in NMO patients, we initially tested proliferation of PBMC to a library of 32 synthetic overlapping 15-mer and 20-mer peptides encompassing the 323-amino acid sequence of full-length human AQP4 (M1 isoform). Here, we studied separate pools containing 5 overlapping AQP4 peptides. By [^3^H]thymidine incorporation, we detected more frequent proliferative responses in primary cultures to AQP4 pools 1–55, 46–100, 126–170, 201–250, and 241–300 (Fig 1A). Lymphocytes from HC also proliferated to some of these pools, and exhibited comparable responses to tetanus toxoid.

Having identified candidate regions of AQP4 containing T-cell determinants, we then tested proliferative responses of NMO patients to individual AQP4 peptides. T-cell determinants were identified within p21–40, p61–80, p131–150, p156–170, and p211–230, which corresponded to intracellular, extracellular, and transmembrane sequences of AQP4 (see Fig 1). Interestingly, 3 of these AQP4 determinants, p61–80, p131–150, and p211–230 are respectively located in extracellular A, C, and E loops, AQP4 domains targeted by NMO-IgG.^14^ The fluorescent dye 5,6-carboxylfluorescein diacetate succinimidyl ester (CFSE) dilution assay is considered a more powerful and sensitive method for detecting proliferation of rare autoantigen-specific human T cells than the traditional [^3^H]thymidine incorporation.^15^ Using this approach, we examined responses to individual AQP4 peptides identified in our initial screening, and also to AQP4 T-cell determinants common to mouse strains with distinct MHC haplotypes.^16^, ^17^ We detected a robust proliferative T-cell response to p61–80, which is located within the extracellular A loop, in all NMO patients tested. T-cell responses were observed to AQP4 p21–40, p156–170, p11–30, and p261–280, although we did not detect substantial proliferation to the latter 2 peptides in our initial [^3^H]thymidine incorporation assays. T cells from HC also recognized these AQP4 peptides, but again, the proliferative responses were both lower and less frequent than in NMO patients. Proliferating AQP4-specific T cells were predominantly CD4^+^, and the proportion of CD4^+^ T cells that responded to AQP4 p61–80 was higher in NMO patients than HC.

**FIGURE 2:**
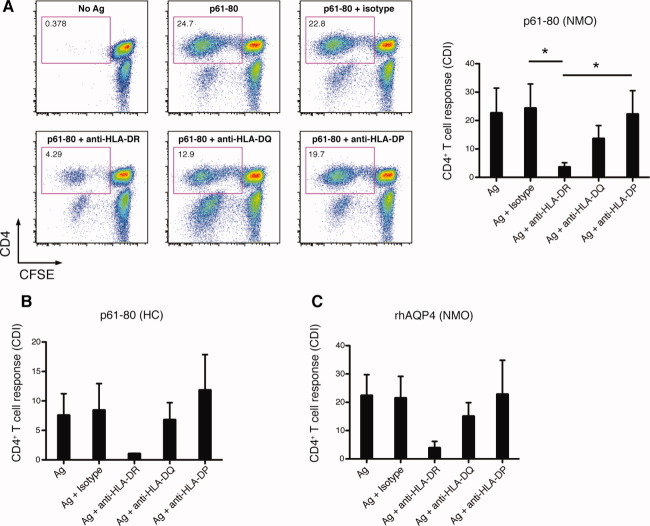
Human leukocyte antigen (HLA)-DR serves as a restriction element for aquaporin 4 (AQP4)-specific T cells. (A, B) 5,6-carboxylfluorescein diacetate succinimidyl ester (CFSE)-labeled peripheral blood mononuclear cells (PBMC) from neuromyelitis optica (NMO) patients were cultured for 10 days with antigens (Ag) alone or in combination with antibodies against HLA-DR, HLA-DQ, or HLA-DP or isotype control antibodies. T-cell proliferation was evaluated by flow-activated cell sorting analysis of CFSE dilution. Inhibitory effects of blocking antibodies were examined on proliferating CD4^+^ T cells (n = 7 NMO in A and n = 4 NMO in B). T-cell proliferation is expressed as cell division index (CDI). (C) PBMC from healthy controls (HC) were similarly examined after stimulation with AQP4 p61–80 (n = 2). Error bars represent standard error of the mean. *p < 0.05, Mann–Whitney *U* test.

Presentation of native protein antigens by APC generally requires proteolytic processing.^18^–^21^ Therefore, we examined whether the AQP4 peptides we identified contained natural T-cell determinants of intact AQP4. When T cells initially stimulated with rhAQP4 were tested for recall responses to individual AQP4 peptides, we observed proliferation to AQP4 p21–40 and p61–80, indicating that these are naturally processed determinants of AQP4 (see Fig 1F). Among peptides that we examined, AQP4 p61–80 was clearly immunodominant. Several studies have identified over-representation of HLA-DPB1*0501, HLA-DRB1*0301, or HLA-DRB3 in NMO patients,^22^–^24^ suggesting that these MHC II alleles could serve as restriction elements for CD4^+^ T cells in NMO. We also identified a high representation of these HLA alleles in our patient cohort; in particular, we noted an over-representation of HLA-DRB3*0202 among NMO subjects (Table). Using MHC II-blocking antibodies, we observed that T-cell proliferative responses to AQP4 p61–80 were inhibited by anti–HLA-DR, but were not statistically inhibited by anti–HLA-DQ or anti–HLA-DP, demonstrating that HLA-DR molecules serve as restriction elements for T cells that recognize this determinant (Fig 2). Proliferation of AQP4 p61–80-specific T cells from HLA-matched HC (see Table) was also inhibited by anti–HLA-DR antibodies. Furthermore, a similar MHC II-restriction profile was observed after stimulating T cells from NMO patients with rhAQP4, suggesting that other AQP4 determinants may also be restricted by HLA-DR molecules.

**TABLE:** Human Leukocyte Antigen Haplotypes of NMO Patients and Healthy Controls

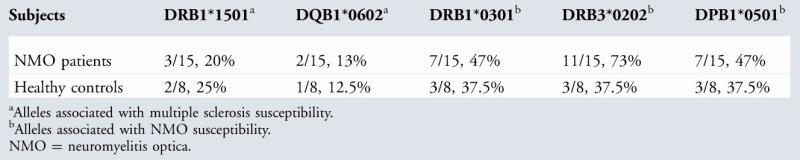

### AQP4 p63–76-Specific T Cells Cross-React with *C. perfringens* ABC-TP p204–217

To characterize the fine specificity of AQP4 p61–80-specific T cells, we examined proliferation to truncated peptides corresponding to sequences within this region. AQP4 p61–80-specific T cells proliferated in response to p61–78 and p61–76, but not to p61–74 or p61–72 (see Fig 3). Shorter AQP4 peptides truncated from the N-terminal sequence, p65–80, p67–80, and p69–80, also stimulated proliferation of p61–80-specific T cells, although less efficiently than p63–80. Collectively, these findings indicated that p63–76 contained the core determinant of AQP4 61–80. In this regard, we observed that p61–80-specific T cells responded nearly as efficiently to p63–76 as to p61–80. Interestingly, AQP4 63–76 contains the predicted binding motif for HLA-DRB1*0301 and HLA-DRB3*0202.

Immune responses to pathogens may elicit cross-reactivity to self-antigens that share structural or sequence homology.^25^, ^26^ This process, known as *molecular mimicry*, is considered an important potential mechanism in autoimmunity. Having found that p63–76 contains an immunodominant AQP4 T-cell epitope, we addressed whether this sequence might share homology with other proteins. We identified 90% homology between AQP4 66–75 and the 10-amino acid sequence 207–216 within conserved ABC-TP proteins from several strains of the bacterium *C. perfringens* (NCBI protein reference sequences ZP_02952885.1, ZP_02638213.1, ZP_02634520.1, ZP_02630305.1; 90% positives, 90% identities, 0% gaps; see Fig 3). T cells from NMO patients proliferated significantly to ABC-TP p204–217, although less intensely than to AQP4 p61–80 and AQP4 p63–76. To directly test for cross-reactivity, T cells initially stimulated with AQP4 p63–76 or ABC-TP were tested for recall responses in a reciprocal manner. Importantly, AQP4-primed T cells proliferated to ABC-TP p204–217 and vice versa, supporting molecular mimicry between this bacterial transmembrane protein and AQP4. Confirming specificity of those recall responses, we did not observe proliferation to AQP4 p156–170.

### AQP4 p61–80-Specific T Cells from NMO Patients Exhibit Proinflammatory Th17 Polarization

**FIGURE 4:**
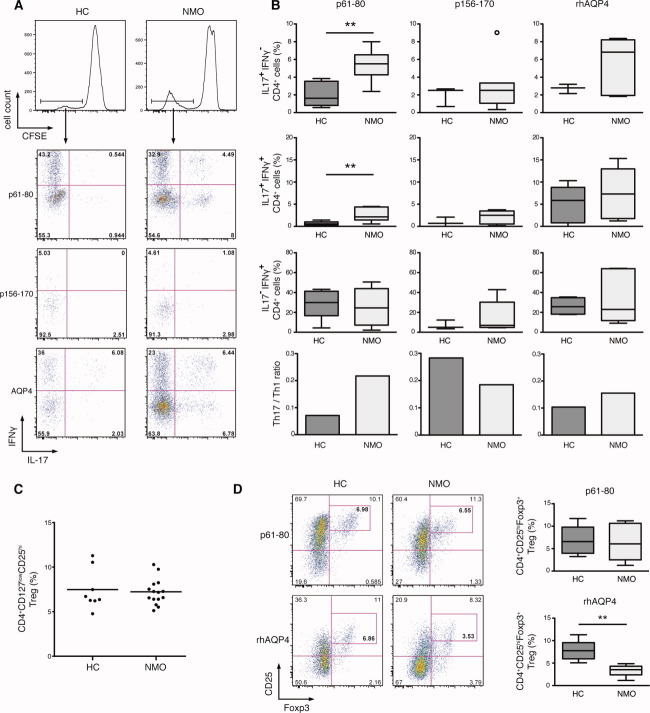
Aquaporin 4 (AQP4) p61–80-specific T cells exhibit a proinflammatory bias. Peripheral blood mononuclear cells (PBMC) were stained with 5,6-Carboxylfluorescein diacetate succinimidyl ester (CFSE) and cultured for 10 days with AQP4 peptides (10μg/ml) or recombinant human (rh) AQP4 (5μg/ml). (A) CD4^+^CFSE^low^ proliferating T cells were analyzed for interleukin (IL)-17 and interferon (IFN)-γ production by intracellular staining after stimulation with phorbol 12-myristate 13-acetate/Ionomycin for 5 hours. (B) Frequencies of IL17^+^IFN-γ^−^, IL17^+^IFN-γ^+^, and IL17^−^IFN-γ^+^ were examined among proliferating p61–80-specific CD4^+^ T cells (n = 8 NMO and n = 5 healthy controls [HC]), p156–170-specific CD4^+^ T cells (n = 6 NMO and n = 3 HC), and rhAQP4-specific CD4^+^ T cells (n = 6 NMO and n = 5 HC). Frequencies of IL-17 and IFN-γ single positive T cells were used to calculate Th17/Th1 ratio. (C) PBMC were examined by fluorescence-activated cell sorting (FACS) for expression of regulatory T cells (Treg)markers including CD4, CD127, and CD25. (D) CFSE-labeled PBMC were cultured for 10 days with AQP4 p61–80 (10μg/ml) or rhAQP4 (5μg/ml). Proliferating CD4^+^ T cells (cell division index > 2) were examined by FACS for expression of CD25^high^, defined as the top half of CD25^+^ cells, and Foxp3 (n = 8 NMO p61–80, n = 6 HC p61–80, n = 7 NMO rhAQP4, and n = 5 HC rhAQP4). Box and whisker plots include the median, distribution, and range. **p < 0.01 Mann–Whitney *U* test.

Although indirect, some clinical and histologic data suggest that Th17 cells may participate in NMO pathogenesis.^6^, ^27^ Thus, we examined proinflammatory cytokine production in proliferating AQP4-specific T cells. In comparison to HC, we observed significantly higher frequencies of IL-17^+^ single- and IL-17^+^IFN-γ^+^ double-positive cells that recognized p61–80 in NMO patients (Fig 4). An increased frequency of Th17 cells from NMO patients was observed after stimulation with rhAQP4, but was not significant. No Th17 bias was detected in response to AQP4 p156–170, suggesting that the Th17 polarization may be epitope specific. In contrast, IFN-γ production by AQP4-specific T cells appeared unchanged between the 2 groups. Thus, the Th17/Th1 ratio was elevated in NMO patients in response to the immunodominant determinant AQP4 p61–80, but not to the other antigens tested. Interestingly, we did not detect a difference in the frequency of peripheral blood regulatory T cells (Treg) from NMO patients and HC. By contrast, the examination of AQP4-specific T cells revealed a significantly reduced frequency of Treg in NMO patients in response to rhAQP4, but not to p61–80.

### Monocytes from NMO Patients Exhibit Proinflammatory Polarization

**FIGURE 5:**
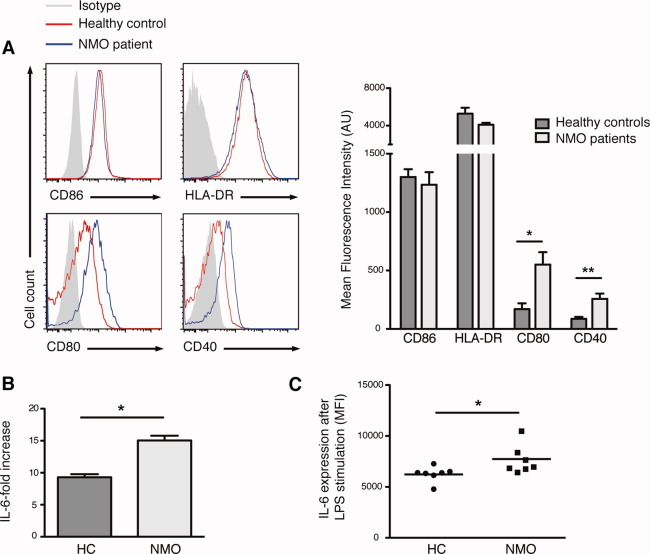
CD14^+^ monocytes from neuromyelitis optica (NMO) patients exhibit increased expression of certain costimulatory molecules and production of interleukin (IL)-6. (A) Peripheral blood mononuclear cells (PBMC) were rested for 4 hours at 37°C. Expression of costimulatory (CD80, CD86, and CD40) and major histocompatibility complex class II molecules was analyzed by flow-activated cell sorting gating on the CD14^+^ population (n = 8 NMO and n = 8 healthy controls [HC]). (B, C) PBMC were stimulated with LPS lipopolysaccharide (LPS; 1μg/ml) for 4 hours. Expression of IL-6 in CD14^+^ monocytes was analyzed by intracellular cytokine staining, before and after LPS stimulation. In C, horizontal lines indicate mean values; in A and B, error bars represent standard error of the mean. *p < 0.05, **p < 0.01 Mann–Whitney *U* test. MFI = mean fluorescent intensity.

APC, including monocytes and other myeloid cells, express costimulatory molecules and secrete specific cytokines that participate in activation and promote lineage commitment of antigen-specific T cells. In this regard, IL-6 is critical for Th17 differentiation.^28^ Previous studies have indicated that serum IL-6 levels are elevated in NMO patients.^7^ As we observed that AQP4 p61–80-specific T cells from NMO patients exhibited Th17 polarization, we questioned whether there were alterations in expression of costimulatory molecules or increased production of IL-6 by myeloid APC. In comparison to HC, there was no evident change in frequency of peripheral blood monocytes (Supplementary Fig). However, we observed increased expression of CD40 and CD80 (Fig 5A), costimulatory molecules that can be associated with proinflammatory T-cell polarization.^29^, ^30^ The frequency of IL-6–producing monocytes was similar in NMO patients and HC. Nevertheless, there were both relative and absolute increases of intracellular IL-6 production after LPS stimulation in monocytes from NMO patients (see Fig 5B, C). No such differences were observed in expression of IL-1β and IL-10. These results indicate that in addition to the known involvement of adaptive immunity, phenotypic changes of cells within the innate immune system may also contribute to NMO pathogenesis.

## Discussion

In this report, we have demonstrated for the first time that peripheral blood T cells from NMO patients and HC proliferate in response to intact AQP4 and AQP4 peptides. However, the frequency and magnitude of T-cell responses to AQP4 determinants was greater in NMO patients. Expansion of those autoreactive T cells provides further evidence that AQP4 is the autoantigen in NMO. It is notable that 3 of the AQP4 T-cell determinants, p61–80, p131–150, and p211–230 are respectively located in extracellular A, C, and E loops, AQP4 domains targeted by NMO-IgG.^14^ Whereas antibodies that target membrane proteins frequently bind conformational determinants exposed on their extracellular surfaces,^31^ CD4^+^ T cells, which are restricted by HLA-D molecules, recognize linear processed peptides that can originate from extracellular, transmembrane, or intracellular domains.^21^ Thus, T and B cells may recognize distinct epitopes of the same autoantigen.^32^, ^33^ Nevertheless, with regard to the B–T collaboration required for IgG production, it is intriguing that p61–80, a naturally processed immunodominant AQP4 T-cell determinant, also represents a target for pathogenic AQP4-specific antibodies.^14^

The frequency of AQP4 p61–80-specific Th17 cells was significantly elevated in NMO patients, a finding that suggests that these autoantigen-specific T cells are a source of IL-17 that drives immunopathogenesis in NMO. In this regard, it was recently demonstrated that Th17 cells more efficiently drive naive B cells to secrete Ig than Th1 cells.^34^ Although our study relates to the peripheral immune response in NMO, our finding that AQP4-specific T cells exhibit a Th17 bias may also be relevant to development of CNS inflammation in NMO. Although neutrophils and eosinophils comprise the predominant cell types within NMO lesions, T cells are also detected.^6^, ^35^ As it is recognized that NMO IgG alone does not induce CNS inflammation and Th17 cells can promote tissue accumulation of neutrophils, AQP4-specific T cells may be sentinel adaptive immune cells directing CNS inflammation in NMO. Via IL-17 secretion, Th17 cells may compromise integrity of the BBB,^36^, ^37^ promote endothelial activation, and stimulate transendothelial migration of neutrophils.^38^ Thus, AQP4-specific Th17 cells may participate in multiple steps of NMO pathogenesis.

Many genetic and environmental factors may contribute to the development of autoimmunity. There is increasing evidence that commensal and pathogenic gut microbiota alter susceptibility to multiple sclerosis (MS), rheumatoid arthritis, type I diabetes, and systemic lupus erythematosus.^39^, ^40^ In this report, we observed a striking sequence homology between the AQP4 T-cell epitope p63–76, which contains predicted binding motifs for 2 NMO-associated HLA-DR molecules,^41^ and p204–217 of a *C. perfringens* ABC-TP. *C. perfringens* is a ubiquitous gram-positive spore-forming bacterium found in human commensal gut flora and also includes specific strains frequently associated with enterotoxin-mediated food poisoning.^42^ We observed T-cell reactivity to ABC-TP p204–217 in peripheral blood of NMO patients, as well as cross-reactivity between it and AQP4 p63–76. Interestingly, we detected 60 to 70% homology between p63–76 and the expressed or predicted ABC-TP in other *Clostridium* species, including the commensal bacteria *C. scindens* and *C. hylemonae* as well as the pathogenic strain *C. sporogenes*. Thus, molecular mimicry could account for T-cell reactivity to AQP4. Besides molecular mimicry, microbes can also exploit innate mechanisms that stimulate proinflammatory or anti-inflammatory immune responses. Recently, it was observed that commensal *Clostridium*-related species alter the balance between Th17 and Treg in mice, and can influence development of autoimmunity.^43^, ^44^ We hypothesize that a *Clostridium* species may have dual functions in NMO pathogenesis, (1) exposing a determinant that cross-reacts with self-antigen; and (2) serving as its own proinflammatory adjuvant, promoting Th17 polarization. This demonstration of molecular mimicry may stimulate further investigation of the potential role of *Clostridium* species in NMO pathogenesis.

Based upon the presumption that the AQP4-specific antibodies of NMO IgG are pathogenic, approaches that reduce humoral immunity, including plasmapheresis, intravenous IgG, and CD20 B-cell depletion, are commonly used in NMO therapy. Although favorable responses to those treatments have been reported, they are often incomplete.^45^ Recognition that AQP4-specific antibodies are T cell-dependent, and alone are not pathogenic in the absence of CNS inflammation, suggests that therapies directed against the cellular arm of NMO pathogenesis could be beneficial. Interestingly, IFN-β, an approved MS therapy that alters cellular immune responses and may influence proinflammatory Th17 activity,^46^ has provoked NMO exacerbations.^47^–^49^ Our observation that T cells specific for the immunodominant AQP4 epitope exhibit Th17 polarization support testing of agents that target the IL-17 axis in NMO.

Collectively, our data provide a foundation to address the potential role of AQP4-specific T cells in driving adaptive humoral and cellular immune responses in NMO pathogenesis. Our observations provide a possible connection between gastrointestinal microbiota, Th17 polarization, and molecular mimicry in the development of CNS autoimmunity.

## Supplementary material

Supplementary Figure. Analysis of CD14+ monocytes from NMO patients and HC. (A) PBMC were examined by FACS for the expression of CD14. (B) PBMC were stimulated with LPS (1 µg/ml) for 4 h or 10 h. (B) Frequencies of IL-10-, IL-1β- and IL-6-positive monocytes after LPS stimulation are shown. (C) Expression of IL-10 and IL-1β in CD14+ monocytes was analyzed by ICS. Horizontal lines indicate mean values.
