# Supplementary figures and images for: Aquaporin 4-Specific T Cells in Neuromyelitis Optica Exhibit a Th17 Bias and Recognize Clostridium ABC Transporter
Source: Ann Neurol. 2012 Jul 17;72(1):53–64. doi: 10.1002/ana.23651 (PMC3405197; doi:10.1002/ana.23651)

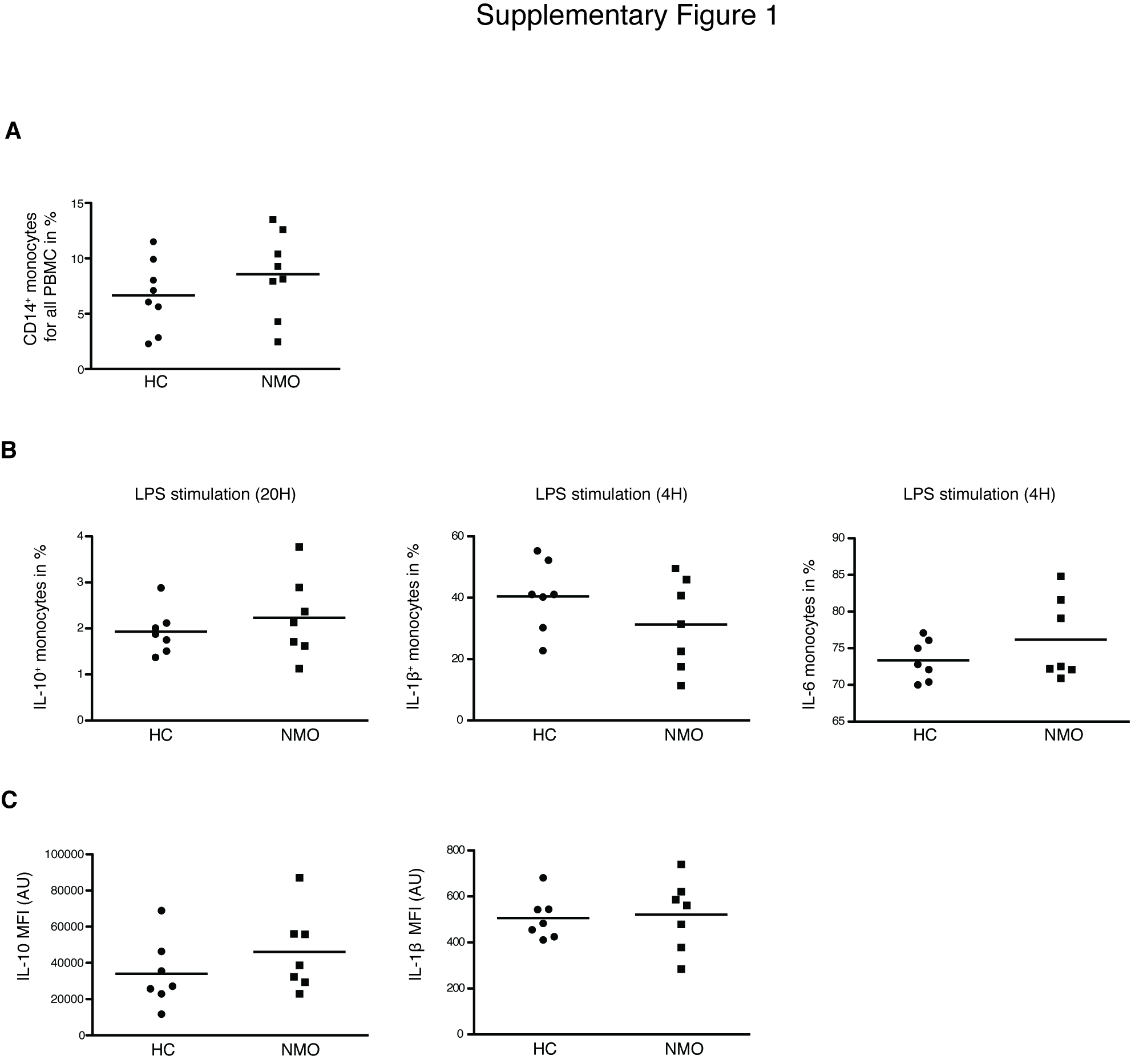

Supplement: Supplementary file 1 [file ana0072-0053-SD1.tif]
