# Supplementary material for: Aquaporin 4-Specific T Cells in Neuromyelitis Optica Exhibit a Th17 Bias and Recognize Clostridium ABC Transporter
Source: Ann Neurol. 2012 Jul 17;72(1):53–64. doi: 10.1002/ana.23651 (PMC3405197; doi:10.1002/ana.23651)
Supplement: Supplementary file 2 [file ana0072-0053-SD2.doc]

**Supplementary Material**

| **Pool** | **Peptide** | **Residues** | **Sequence** |  |
| --- | --- | --- | --- | --- |
| **1** | **p1** | 1-20 | **MSDRPTARRWGKCGPLCTRE** |  |
|  | **p11** | 11-30 | **GKCGPLCTRENIMVAFKGVW** |  |
|  | **p21** | 21-40 | **NIMVAFKGVWTQAFWKAVTA** |  |
|  | **p31** | 31-50 | **TQAFWKAVTAEFLAMLIFVL** |  |
|  | **p41** | 41-55 | **EFLAMLIFVLLSLGS** |  |
| **2** | **p46** | 46-60 | **CTRKISIAKSVFYIA** |  |
|  | **p51** | 51-70 | **LSLGSTINWGGTEKPLPVDM** |  |
|  | **p61** | 61-80 | **GTEKPLPVDMVLISLCFGLS** |  |
|  | **p71** | 71-90 | **VLISLCFGLSIATMVQCFGH** |  |
|  | **p81** | 81-100 | **IATMVQCFGHISGGHINPAV** |  |
| **3** | **p91** | 91-110 | **ISGGHINPAVTVAMVCTRKI** |  |
|  | **p101** | 101-115 | **TVAMVCTRKISIAKS** |  |
|  | **p106** | 106-120 | **CTRKISIAKSVFYIA** |  |
|  | **p111** | 111-130 | **SIAKSVFYIAAQCLGAIIGA** |  |
|  | **p121** | 121-135 | **AQCLGAIIGAGILYL** |  |
| **4** | **p126** | 126-140 | **AIIGAGILYLVTPPS** |  |
|  | **p131** | 131-150 | **GILYLVTPPSVVGGLGVTMV** |  |
|  | **p141** | 141-160 | **VVGGLGVTMVHGNLTAGHGL** |  |
|  | **p151** | 151-165 | **HGNLTAGHGLLVELI** |  |
|  | **p156** | 156-170 | **AGHGLLVELIITFQL** |  |
|  |  |  |  |  |

**Supplementary Table**. Human AQP4 peptide library.

| **Pool** | **Peptide** | **Residues** | **Sequence** |  |
| --- | --- | --- | --- | --- |
| **5** | **p161** | 161-175 | **LVELIITFQLVFTIF** |  |
|  | **p171** | 171-190 | **VFTIFASCDSKRTDVTGSIA** |  |
|  | **p181** | 181-200 | **KRTDVTGSIALAIGFSVAIG** |  |
|  | **p191** | 191-205 | **LAIGFSVAIGHLFAI** |  |
|  | **p196** | 196-210 | **SVAIGHLFAINYTGA** |  |
| **6** | **p201** | 201-220 | **HLFAINYTGASMNPARSFGP** |  |
|  | **p211** | 211-230 | **SMNPARSFGPAVIMGNWENH** |  |
|  | **p221** | 221-235 | **AVIMGNWENHWIYWV** |  |
|  | **p226** | 226-240 | **NWENHWIYWVGPIIG** |  |
|  | **p231** | 231-250 | **WIYWVGPIIGAVLAGGLYEY** |  |
| **7** | **p241** | 241-260 | **AVLAGGLYEYVFCPDVEFKR** |  |
|  | **p251** | 251-270 | **VFCPDVEFKRRFKEAFSKAA** |  |
|  | **p261** | 261-280 | **RFKEAFSKAAQQTKGSYMEV** |  |
|  | **p271** | 271-290 | **QQTKGSYMEVEDNRSQVETD** |  |
|  | **p281** | 281-300 | **EDNRSQVETDDLILKPGVVH** |  |
| **8** | **p291** | 291-310 | **DLILKPGVVHVIDVDRGEEK** |  |
|  | **p301** | 301-320 | **VIDVDRGEEKKGKDQSGEVL** |  |
|  | **p304** | 304-323 | **VDRGEEKKGKDQSGEVLSSV** |  |
|  |  |  |  |  |

**Supplementary Figure Legend.**

**Supplementary Figure. Analysis of CD14^+^ monocytes from NMO patients and HC. (**A) PBMC were examined by FACS for the expression of CD14. (B) PBMC were stimulated with LPS (1 μg/ml) for 4 h or 10 h. (B) Frequencies of IL-10-, IL-1β- and IL-6-positive monocytes after LPS stimulation are shown. (C) Expression of IL-10 and IL-1β in CD14^+^ monocytes was analyzed by ICS. Horizontal lines indicate mean values.
